# Neighborhood Competition Correlates with Height–Diameter Growth Allocation and Gene Expression in Clonal *Eucalyptus urophylla* × *E. grandis* Plantation

**DOI:** 10.3390/plants15213341

**Published:** 2026-10-28

**Authors:** Yongyu Yang, Qingfen Li, Caixiong Chen, Ziqiao Ke, Cheng Deng

**Affiliations:** 1 College of Forestry and Landscape Architecture, South China Agricultural University, Guangzhou 510642, China; yangyongyu1@outlook.com (Y.Y.); ltlyz2014@163.com (C.C.); keziqiao2002@163.com (Z.K.); 2 Guangdong Provincial Key Laboratory of Forest Plant Germplasm Innovation and Utilization, Guangzhou 510642, China

**Keywords:** *Eucalyptus*, height–diameter allometry, intra-stand competition, WGCNA, qRT-PCR

## Abstract

Height–diameter (H–D) growth patterns describe the relative growth of tree height and stem diameter and may vary with local competitive conditions. We examined a *Eucalyptus urophylla* × *E. grandis* clone DH32-29 plantation across 12 stand units. Phenotypic comparisons included 364 interior trees and 254 true stand-edge trees; RNA-seq was conducted on a balanced subset of 72 trees (36 interior and 36 edge trees), not on the full phenotypic sample. Neighborhood competition around interior trees was characterized using the Hegyi index and basal area in larger trees (BAL), and growth traits were compared between interior and edge trees. Hegyi and BAL were negatively associated with growth, with stronger associations for diameter increment than for height increment. Unadjusted comparisons suggested different growth patterns, but plot-adjusted sensitivity analysis supported differences only in diameter increment and H/D. WGCNA identified modules associated with individual allometric traits, and XGBoost prioritized expression features with moderate model performance (*R*^2^ = 0.404). qRT-PCR supported expression trends for selected genes but did not test gene function. These observational results identify associations among neighborhood structure, growth patterns, and gene-expression variation; edge contrasts may also reflect differences in light, wind exposure, microclimate, or soil conditions. The candidate modules and genes require independent and functional validation.

## 1. Introduction

Growth allocation between height extension and stem thickening is a central component of plant development. It influences tree architecture, mechanical support, biomass accumulation, and performance within a stand [1,2,3,4]. This balance is especially relevant in fast-growing *Eucalyptus* plantations, which supply wood and fiber across tropical and subtropical regions and are commonly established with genetically uniform material [5,6,7,8]. Understanding how individual trees adjust height and diameter growth within these stands can therefore inform both plant-growth biology and plantation assessment.

The height–diameter (H–D) relationship provides a tractable measure of growth allocation because it integrates vertical extension and radial growth. H–D allometry varies among species and populations and responds to climate, site conditions, stand development, and management [9,10,11,12]. In *Eucalyptus*, variation in H–D traits is relevant to stem form, stand stability, and productivity [13,14,15,16]. However, stand-level relationships can obscure differences among neighboring trees. Individual-tree analyses are needed to determine how local competitive conditions are associated with the relative allocation of growth to height and diameter.

Competition is spatially heterogeneous even within an even-aged plantation. Neighbor size, distance, and local crowding may constrain access to growing space and resources, and their associations with height and diameter growth need not be equivalent [14,17,18]. The Hegyi competition index and basal area in larger trees (BAL) describe complementary aspects of a focal tree’s competitive neighborhood, while neighborhood tree number and crown overlap characterize local stand structure [15,19,20,21]. Trees in the stand interior and trees at a true plantation edge also experience contrasting neighborhood geometry. An edge position is not an unconfounded low-competition reference: edge trees may also differ in light, wind exposure, microclimate, and soil conditions. Therefore, center–edge contrasts can describe field associations but cannot isolate the effect of neighborhood competition from other edge-related factors.

The molecular processes accompanying competitive differences in tree growth remain less clear. Stem development involves coordinated changes in cambial activity, carbon metabolism, stress responses, and gene expression [22,23,24,25]. RNA sequencing and weighted gene co-expression network analysis (WGCNA) can identify expression modules associated with quantitative growth traits [26,27,28]. Machine-learning methods can then prioritize candidate features, and qRT-PCR can assess whether selected RNA-seq expression trends are reproducible [29,30]. Integrating these approaches with individual-tree measurements may reveal transcriptional correlates of allometric variation, but the resulting associations require functional validation.

Here, we examined a clonal *Eucalyptus urophylla* × *E. grandis* plantation to determine how local competitive heterogeneity relates to H–D growth patterns and gene expression. We hypothesized that (i) stronger neighborhood competition would be associated more strongly with reduced diameter increment than with reduced height increment; (ii) interior trees would exhibit a more height-oriented growth pattern than true stand-edge trees; and (iii) co-expression modules would covary with individual-level allometric traits, while qRT-PCR would assess expression trends for selected candidate genes only and would not validate their biological functions. We quantified neighborhood competition and repeated growth, compared interior and edge trees, and integrated RNA-seq, WGCNA, XGBoost, and qRT-PCR analyses. Because the study was observational, conducted at one site with one clone, and used a single cambial-tissue sampling time point, its results are interpreted as associations. In addition, the competition indices do not directly measure light, wind, microclimate, or soil conditions, and edge contrasts may reflect these factors as well as neighborhood structure.

## 2. Results

### 2.1. Competition Intensity Is Associated with Reduced Radial Growth

**Figure 1:**
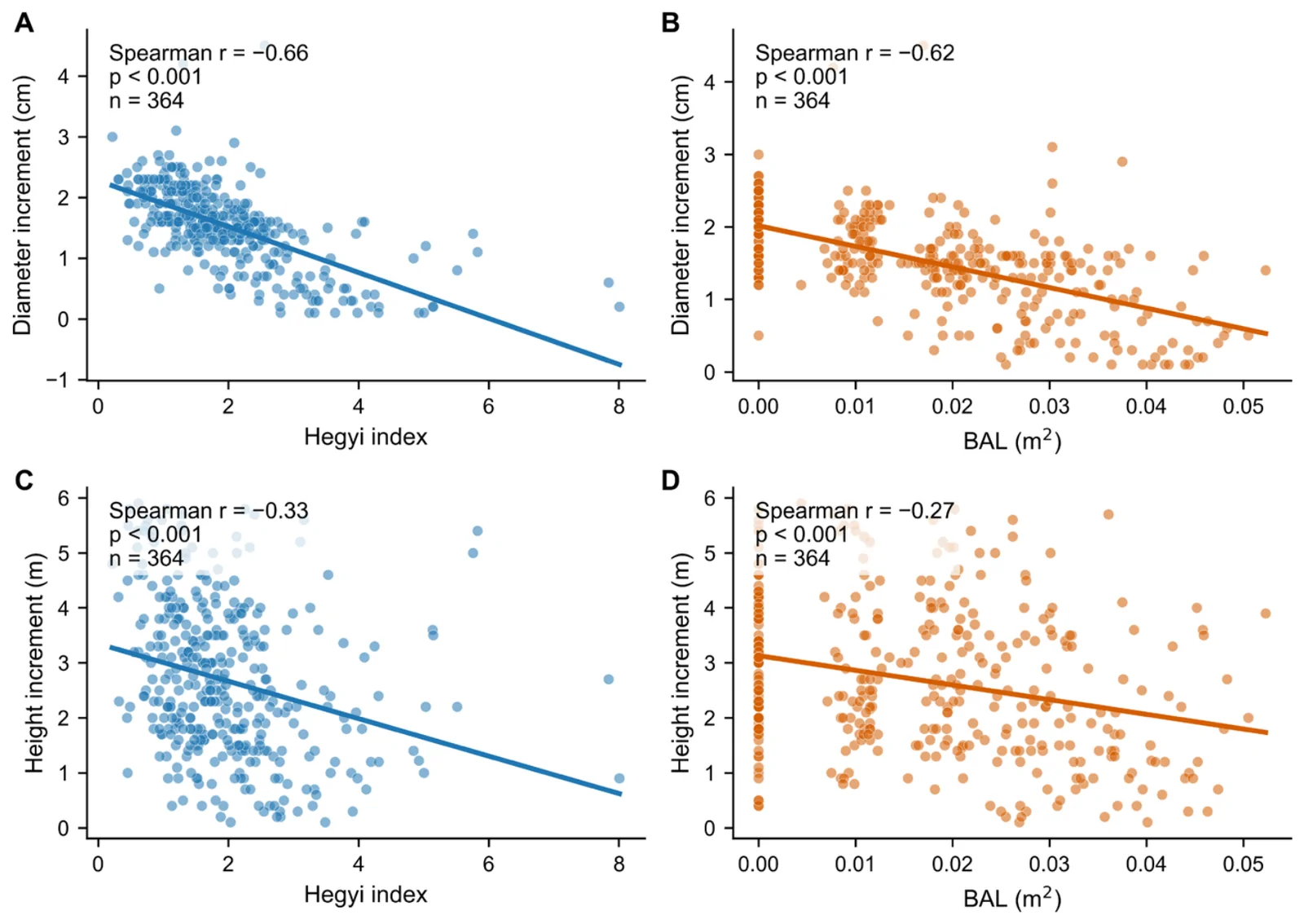
Associations between neighborhood competition indices and growth responses of center trees. Hegyi is a distance-weighted neighborhood competition index; BAL is basal area in larger neighboring trees within the 3 m neighborhood radius. Points represent individual trees, and solid lines represent fitted linear relationships. Spearman correlation coefficients and *p* values are shown in each panel; each panel includes *n* = 364 center trees. Panels show (**A**) Hegyi versus diameter increment; (**B**) BAL versus diameter increment; (**C**) Hegyi versus height increment; and (**D**) BAL versus height increment. Blue denotes Hegyi panels, and orange denotes BAL panels.

Neighborhood competition indices were negatively associated with subsequent tree growth, with stronger relationships for radial growth than for height growth (Figure 1). The Hegyi competition index showed a negative association with diameter increment (Spearman *r =* −0.66, *p* < 0.001) and a weaker negative association with height increment (Spearman *r =* −0.33, *p* < 0.001). BAL showed a similar pattern, with a stronger negative association with diameter increment (Spearman *r =* −0.62, *p* < 0.001) than with height increment (Spearman *r =* −0.27, *p* < 0.001). These results suggest that greater local competitive heterogeneity, as represented by the neighborhood indices, was accompanied primarily by reduced radial growth.

Across the eight prespecified correlations of the four neighborhood indices with diameter and height increment, all four indices were negatively associated with diameter increment (Hegyi: ρ = −0.660, *p* = 6.87 × 10^−47^, q = 5.49 × 10^−46^; BAL: ρ = −0.616, *p* = 2.44 × 10^−39^, q = 9.77 × 10^−39^; neighbor number: ρ = −0.354, *p* = 3.41 × 10^−12^, q = 9.1 × 10^−12^; crown overlap ratio: ρ = −0.286, *p* = 2.9 × 10^−8^, q = 4.65 × 10^−8^). Associations with height increment were also negative (Hegyi: ρ = −0.330, *p* = 1.13 × 10^−10^, q = 2.26 × 10^−10^; BAL: ρ = −0.270, *p* = 1.6 × 10^−7^, q = 2.14 × 10^−7^; neighbor number: ρ = −0.251, *p* = 1.28 × 10^−6^, q = 1.46 × 10^−6^; crown overlap ratio: ρ = −0.138, *p* = 0.00856, q = 0.00856). *p* values were adjusted across these eight correlations using the Benjamini–Hochberg procedure; complete results are provided in Supplementary Table S3. These tree-level analyses were not adjusted for clustering within stand units.

### 2.2. Center and Edge Trees Differ in Growth Allocation

**Figure 2:**
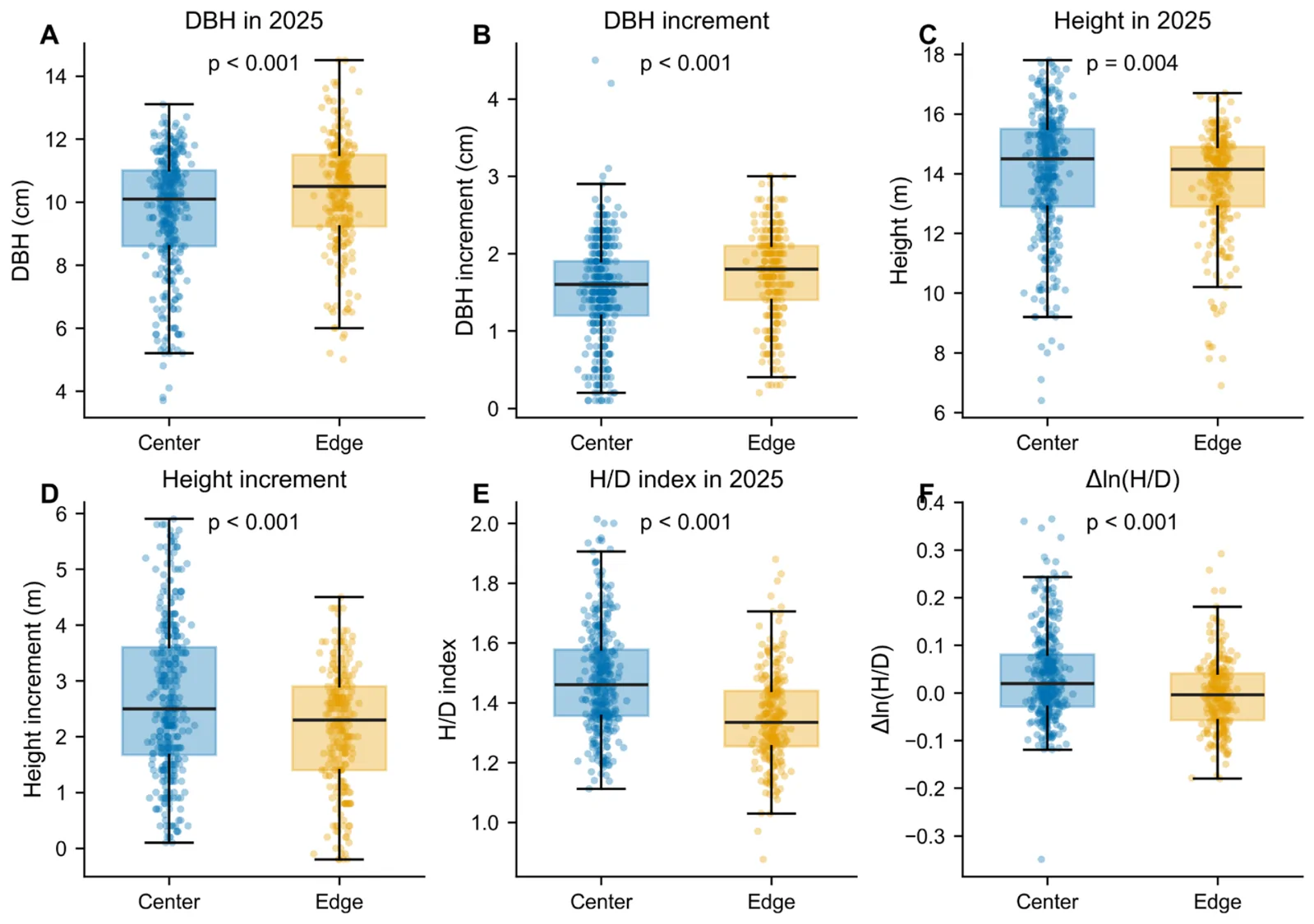
Differences in diameter and height growth between interior and edge trees. Panels (**A**–**F**) show, respectively, DBH in 2025, diameter increment, height in 2025, height increment, H/D index in 2025, and Δ ln(H/D). H/D is height in meters divided by DBH in centimeters, and Δ ln(H/D) is the change in log-transformed H/D across the measurement interval. The figure shows *n* = 364 center trees and *n* = 254 edge trees; panel *p* values are from tree-level Mann–Whitney U tests; plot-adjusted sensitivity results are reported in Supplementary Table S5. Blue represents center trees, and yellow–orange represents edge trees.

Center and edge trees were compared using the full phenotypic dataset (364 center and 254 edge trees; Figure 2). In the unadjusted tree-level comparisons, edge trees had higher median DBH and diameter increment, whereas center trees had higher median height, H/D, and change in ln(H/D). Because trees were clustered within 12 stand units, we additionally fitted models with stand-unit fixed effects and standard errors clustered by unit. In this sensitivity analysis, the edge–center difference remained after Benjamini–Hochberg correction for diameter increment (adjusted difference = 0.182 cm, 95% CI 0.042 to 0.321, q = 0.046) and H/D (adjusted difference = −0.121, 95% CI −0.179 to −0.063, q = 0.0046). The contrasts in baseline DBH, height, height increment, and change in ln(H/D) were not statistically supported after accounting for stand unit (Supplementary Table S5). Thus, the field evidence for center–edge differences is narrower than suggested by unadjusted tree-level tests. RNA-seq was conducted on a subset of 72 sampled trees, not on all phenotyped trees.

### 2.3. WGCNA Identifies Modules Associated with Allometric Growth Traits

**Figure 3:**
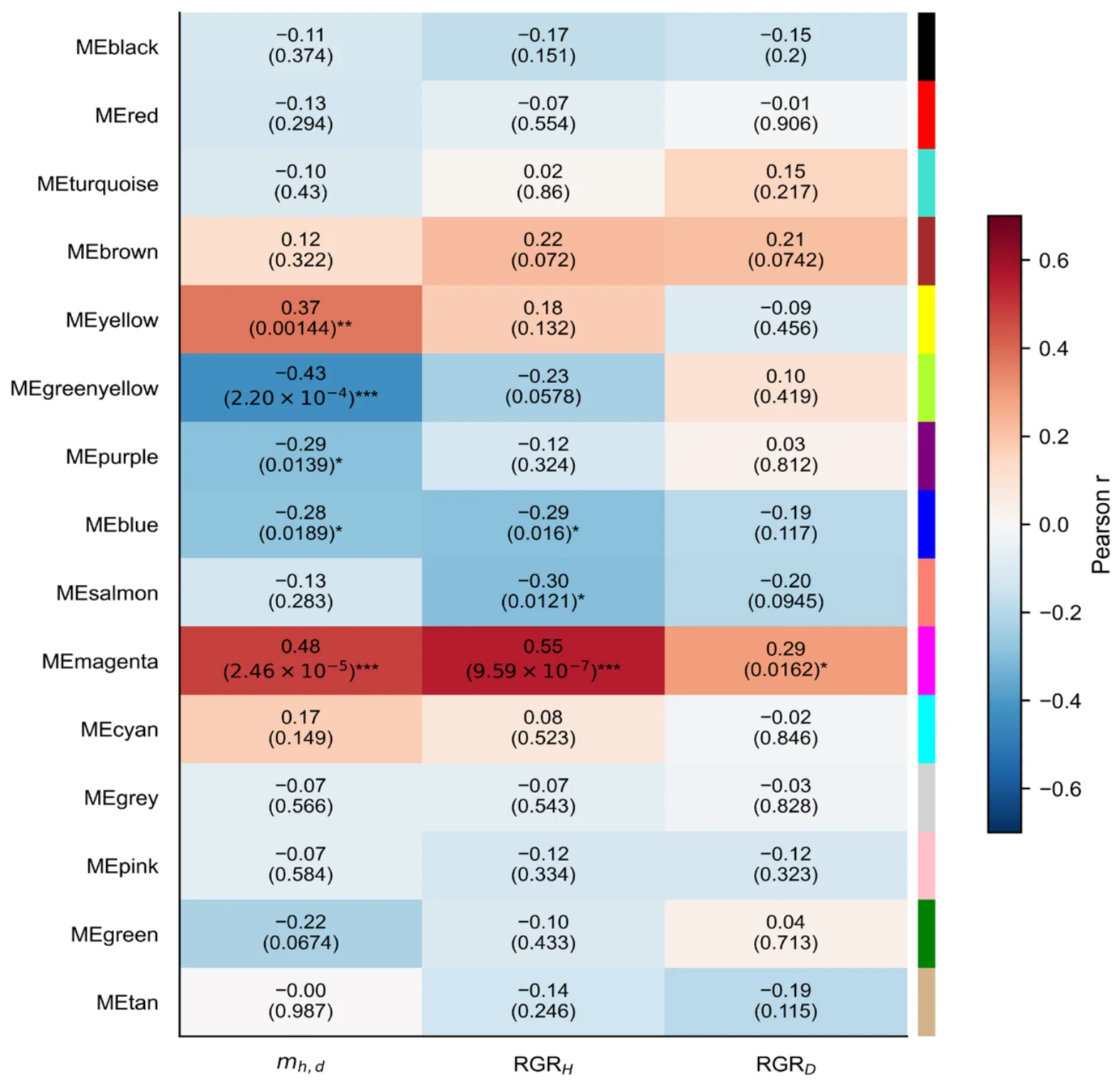
WGCNA module–trait correlations for selected allometric growth traits. The heatmap shows Pearson correlation coefficients between module eigengenes and traits, with nominal *p* values in parentheses. Benjamini–Hochberg-adjusted q values for all 105 tests in the exported 15-module × 7-trait matrix are provided in Supplementary Table S2. *RGR_H_* is relative height growth, *RGR_D_* is relative diameter growth, and *m_h,d_* is the individual-level allometric growth coefficient calculated as *RGR_H_*/*RGR_D_*. The exported WGCNA expression matrix contained 70 samples. Red indicates positive correlations, blue indicates negative correlations, and the side strip identifies module colors. Asterisks indicate nominal p-value thresholds: * *p* < 0.05, ** *p* < 0.01, and *** *p* < 0.001.

The LC-Bio cloud export contained module–trait correlations for 15 modules and seven traits (RATIO, DBH, HEIGHT, *m_h,d_*, *RGR_D_*, *RGR_H_*, and position). We recalculated Benjamini–Hochberg q values across all 105 module–trait tests (Supplementary Table S2). MEmagenta remained positively associated with *m_h,d_* (r = 0.48, nominal *p* = 2.46 × 10^−5^, q = 0.00129) and *RGR_H_* (r = 0.55, nominal *p* = 9.59 × 10^−7^, q = 0.000101), whereas its association with *RGR_D_* was nominal only (r = 0.29, *p* = 0.0162, q = 0.106). The exported matrix did not include Hegyi, BAL, neighborhood number, or crown overlap; therefore, it does not establish direct module–competition-index associations. Selected module–growth-trait correlations are shown in Figure 3.

Differential expression was assessed separately within each of the 12 stand units by comparing three edge samples with three center samples per unit. Using |log2FC| > 1 and adjusted q < 0.05, the unit-specific contrasts identified 93–3908 differentially expressed genes (DEGs) per unit. Across all 12 contrasts, there were 9380 contrast-specific DEG records: 3196 records indicated higher expression at the edge and 6184 indicated higher expression in the center. These records represented 6247 unique genes after deduplication across units (Supplementary Table S1). The WGCNA network was constructed separately from the filtered expression matrix and was not restricted to the DEG lists.

### 2.4. Machine-Learning Analysis Prioritizes Candidate Hub Genes with Moderate Predictive Performance

**Figure 4:**
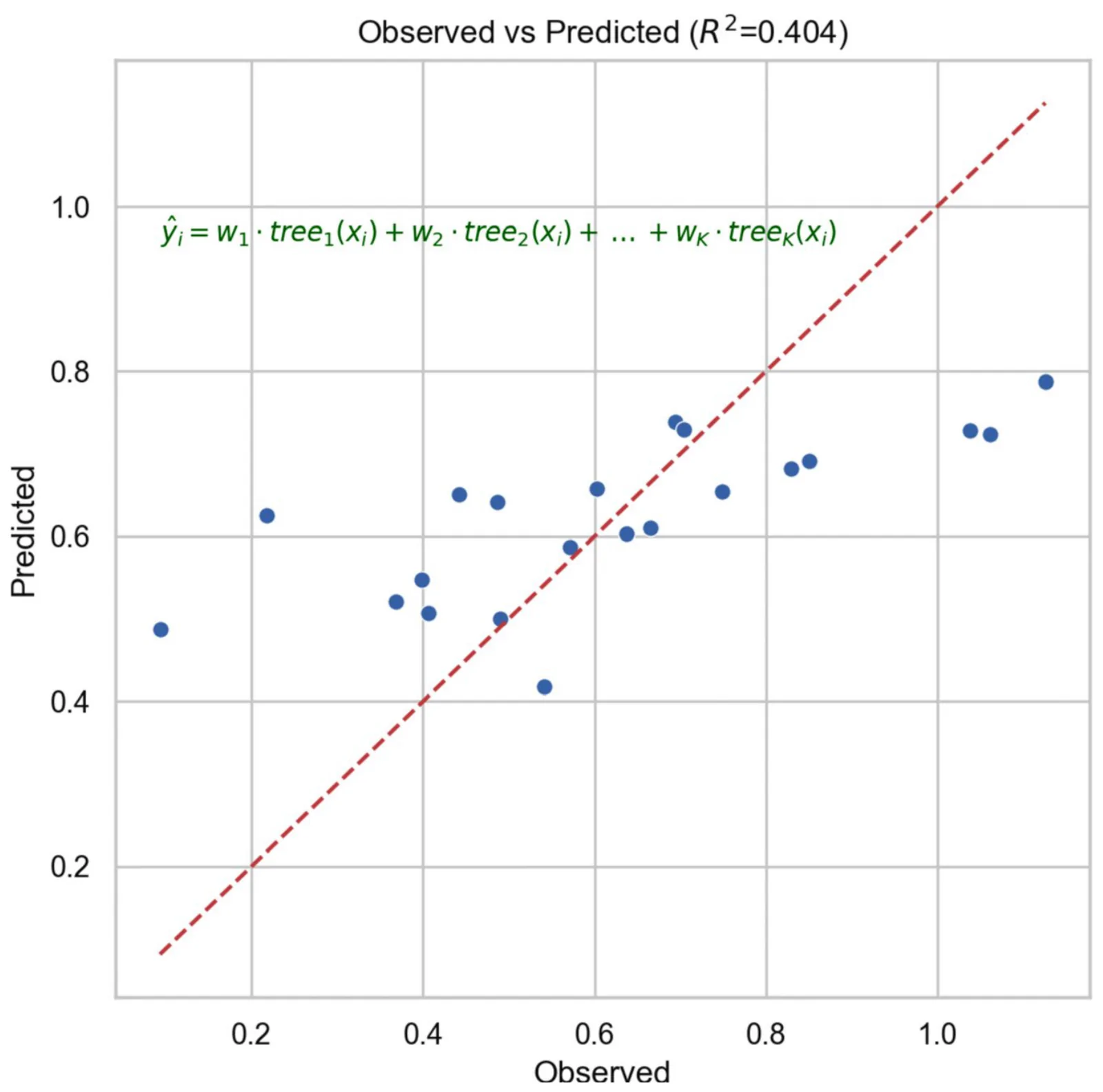
Observed versus predicted individual-level allometric growth coefficient (*m_h,d_* = *RGR_H_*/*RGR_D_*) from the XGBoost model. Points represent the 21 held-out test trees; the dashed line is the 1:1 reference. Test-set performance was *R*^2^ = 0.404, RMSE = 0.201, and MAE = 0.158. The saved feature-importance output listed 175 predictor features.

The saved feature-importance output listed 175 predictor features. The saved model-evaluation file reports a held-out test set of 21 trees and a training set of 49 trees. On the test set, the model achieved *R*^2^ = 0.404, RMSE = 0.201, and MAE = 0.158 (Figure 4), indicating moderate predictive performance and substantial unexplained variation in *m_h,d_*. XGBoost was used to prioritize associated features, not to provide functional evidence or establish regulatory causality.

### 2.5. qRT-PCR Supports RNA-Seq Expression Patterns of Selected Hub Genes

**Figure 5:**
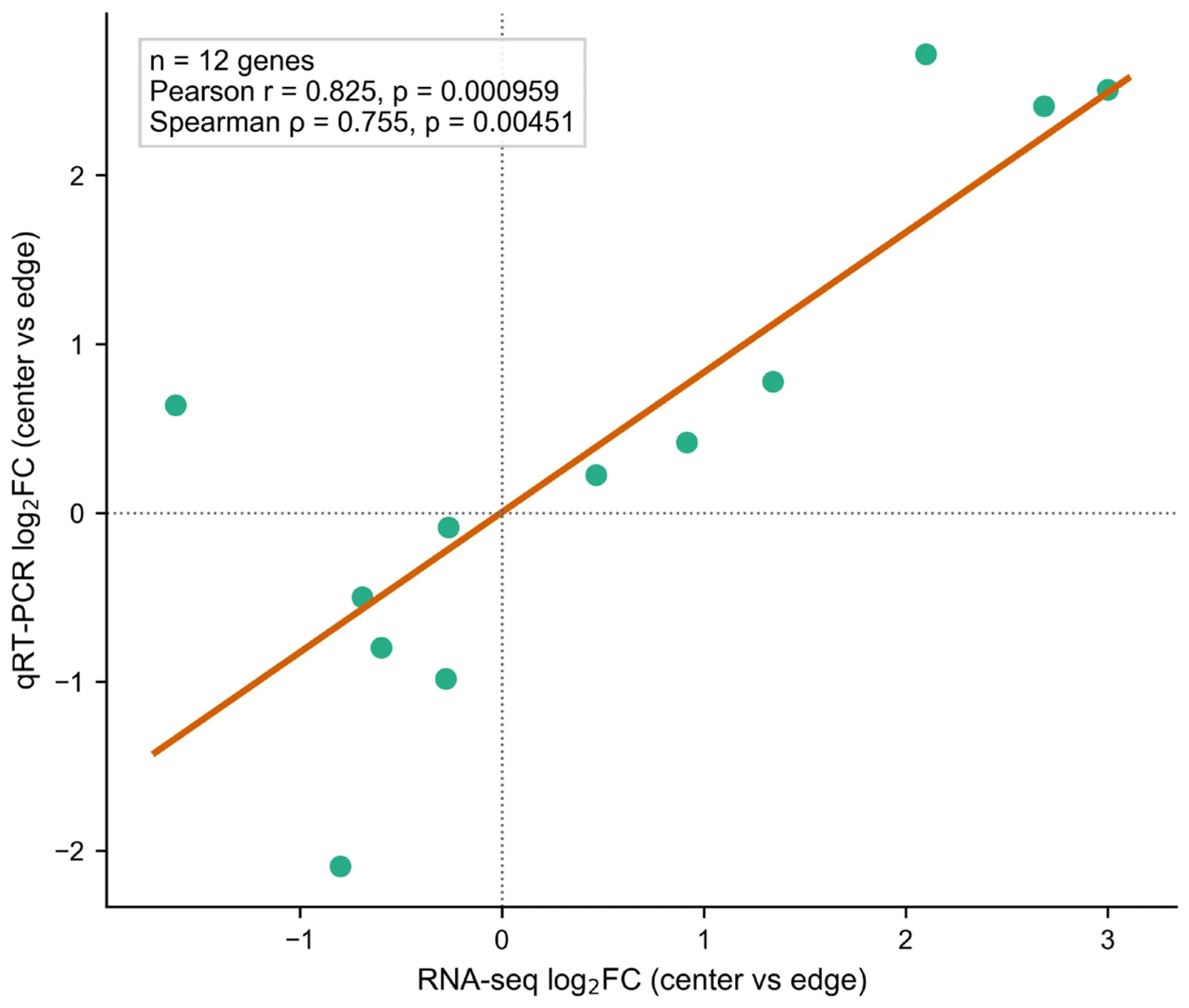
Concordance between RNA-seq and qRT-PCR expression estimates for 12 selected candidate hub genes. The x-axis shows RNA-seq log_2_ fold change for center versus edge trees, and the y-axis shows qRT-PCR log_2_ fold change for the same contrast. Points represent genes; the solid line is the fitted linear relationship. Pearson r = 0.825 (*p* = 0.00096) and Spearman ρ = 0.755 (*p* = 0.0045). These expression-trend comparisons do not provide functional validation.

qRT-PCR supported the RNA-seq expression trends for 12 selected candidate hub genes associated with allometric growth traits (Figure 5). For each gene, RNA-seq log_2_ fold change between center and edge trees was compared with the corresponding qRT-PCR log_2_ fold change using the paired values in the designated qPCR correlation sheet. Across the 12 genes, the association was positive (Pearson r = 0.825, *p* = 0.00096; Spearman ρ = 0.755, *p* = 0.0045). This cross-gene concordance supports the consistency of the expression trends in this candidate panel, but it does not validate gene function.

## 3. Discussion

### 3.1. Competitive Heterogeneity and Divergent Growth Allocation

The competition-growth analyses suggest that local competitive heterogeneity was more closely associated with radial growth than with height growth. All four indices (Hegyi, BAL, neighbor number, and crown overlap ratio) were negatively associated with diameter increment after Benjamini–Hochberg correction across eight tests; associations with height increment were also negative, although generally weaker. This indicates that trees in denser or more overlapping neighborhoods tended to show reduced radial thickening. This pattern is consistent with studies showing that neighborhood competition and stand-density conditions can be associated with variation in tree growth, size structure, and allometric relationships [14,15,18,31]. Because these indices describe neighborhood structure rather than directly measured light, water, nutrients, or wind, this pattern should be interpreted as an association between competitive position and growth allocation rather than as direct environmental causality.

Height–diameter allometry can be interpreted as an adaptive growth strategy that reflects the balance between light acquisition, radial support, hydraulic transport, and survival. Height growth improves canopy access and carbon acquisition, whereas diameter growth contributes to mechanical stability, xylem transport capacity, and resistance to bending or mechanical deformation [32,33]. Trees investing proportionally less in radial reinforcement may achieve faster vertical extension, but this strategy can increase stem slenderness and reduce mechanical safety margins. Therefore, the stronger association of competition indices with diameter increment in the present study suggests that local competitive pressure was closely linked to the balance between vertical extension and radial reinforcement, consistent with adaptive growth-allocation theory and previous evidence that tree allometry varies with species, environment, and stand structure [9,31].

This interpretation is also consistent with size-asymmetric competition, in which larger or better-positioned neighbors can be associated with disproportionate differences in resource acquisition and growth allocation [17]. In this plantation, greater competition was accompanied primarily by lower diameter increment, suggesting that radial growth may be a sensitive field indicator of local competitive pressure. This has practical relevance for fast-growing *Eucalyptus* plantations, where diameter increment, H/D index, and changes in H/D can help evaluate stem slenderness, stand density effects, biomass allocation, and potential trade-offs between height-oriented growth and radial thickening [3,13,16,34,35].

### 3.2. Center–Edge Differences as Field Evidence of Growth-Allocation Plasticity

The center–edge comparison should be interpreted in light of the plot-adjusted sensitivity analysis. After accounting for stand unit and clustering standard errors by unit, the edge–center contrasts in diameter increment and H/D remained supported after correction across the six field traits, whereas contrasts in baseline DBH, height, height increment, and change in ln(H/D) did not. The stronger unadjusted differences therefore partly reflected between-unit structure. Moreover, edge positions can differ in light, wind exposure, microclimate, and soil conditions, so these observations cannot isolate neighborhood competition as the cause of the remaining contrasts.

Earlier allometric studies often treated height–diameter relationships as relatively stable during growth, but increasing evidence indicates that allometric trajectories can change with stand development, management, and competitive conditions [36,37]. In denser or more competitive environments, trees may allocate proportionally more growth to height to obtain or maintain canopy access, whereas reduced stand density or greater lateral growing space can favor radial growth that strengthens support and hydraulic capacity [38]. This framework is consistent with the present center–edge comparison: interior trees showed higher H/D and Δ *ln(H/D)*, whereas true edge trees showed greater DBH and diameter increment. Edge-effect studies provide useful context for boundary-related variation in structure and exposure, but plantation edges should be interpreted as true stand-edge positions rather than fixed-plot boundaries [39,40]. Future measurements of light, wind exposure, soil water, and nutrients would help link these field contrasts more directly to resource and mechanical gradients.

### 3.3. Co-Expression Modules Associated with Allometric Traits

The WGCNA results indicate that allometric variation covaried with coordinated gene-expression patterns. After Benjamini–Hochberg correction across all 105 tests in the available 15-module × 7-trait export, MEmagenta remained positively associated with *m_h,d_* (q = 0.00129) and *RGR_H_* (q = 0.000101); its association with *RGR_D_* was nominal only (q = 0.106). These associations are not evidence of regulatory causality. The exported matrix did not contain the four neighborhood competition indices, so direct module–competition correlations could not be evaluated from this analysis output.

Growth allocation and stem form are quantitative traits shaped by genetic background and modified by environmental and management conditions. Because traits such as tree height, diameter, and H/D-related indices show continuous variation, molecular-marker, QTL, association-mapping, and transcriptomic studies provide useful approaches for identifying genomic regions or genes associated with stem architecture, secondary growth, and vascular cambium activity [41,42,43,44,45,46,47,48,49]. In this context, the MEmagenta module and selected hub genes identified here should be viewed as candidate molecular signals associated with allometric growth variation. These molecular clues complement the field evidence from competition indices and center–edge growth allocation, and they provide a focused basis for future functional tests.

### 3.4. Candidate Hub Genes, XGBoost, and qRT-PCR Validation

The XGBoost model achieved moderate held-out test performance (*R*^2^ = 0.404), indicating that the recorded predictor set captured only part of the variation in *m_h,d_* and that substantial variation remained unexplained. Its feature rankings provide candidates for follow-up, but do not identify causal regulators. Tree-boosting methods can screen complex predictor sets [29], although the model’s predictive performance and feature rankings require cautious interpretation in light of the small test set and the incomplete record of model tuning and feature construction. These results are consistent with transcriptome-based association and systems-genetics approaches in forest trees [44,49].

The qRT-PCR analysis supported the RNA-seq expression trends across selected hub genes, improving confidence in the reliability of the observed expression differences between center and edge trees. This concordance strengthens the transcriptomic screening results and provides a practical shortlist of candidate genes for future functional studies. Controlled seedling experiments, transient transformation, VIGS, gene editing, or other perturbation approaches could further test whether these candidates contribute to height growth, radial growth, or height–diameter allocation, while careful qRT-PCR reporting remains important for reproducibility [30,50].

### 3.5. Limitations and Future Directions

Several limitations are important for interpreting these results. The field comparisons are observational and the center–edge contrast cannot isolate neighborhood competition from edge-related light, wind exposure, microclimate, or soil conditions. The study used 12 stand units; plot-adjusted analyses were added, but spatial autocorrelation was not assessed. The study was conducted at a single site using a single *Eucalyptus* hybrid clone, which limits generalizability across genotypes, stand ages, and plantation environments. Third, RNA-seq was sampled at one time point, limiting interpretation of diel or circadian processes. Repeated temporal sampling across day-night cycles and across growth stages would be needed to evaluate such patterns more directly, particularly because carbon allocation and nonstructural carbohydrate dynamics can vary strongly over time and among tissues [22,25,51].

Additional limitations relate to tissue specificity and statistical structure. Cambial or adjacent phloem tissue provides information relevant to stem growth, but it does not fully represent apical meristems, leaves, roots, or whole-tree height-growth processes. WGCNA and machine learning are association-based approaches, and potential plot-level non-independence or spatial autocorrelation may remain in transcriptomic analyses. Finally, qRT-PCR supported RNA-seq expression trends but did not provide functional validation. These limitations are consistent with broader cautions that plastic responses and resource allocation should be evaluated across environmental contexts, tissues, and time scales [34,35].

Future work should combine direct microclimate and soil monitoring with repeated phenotyping and transcriptomic sampling. Multi-tissue sampling, independent-site validation, and controlled competition, wind, or light experiments would help separate the effects of local competition from other environmental gradients. Functional testing of candidate genes will also be necessary before assigning mechanistic roles. For plantation applications, a practical next step would be to test whether field indicators, such as H/D, Δ *ln(H/D)*, Hegyi, or BAL, can be linked to transcriptomic signatures without requiring transcriptome profiling at operational scale. Temporal sampling would also help evaluate whether sugar-related and stress-associated signals are stable features of growth allocation or transient expression states [23,24].

### 3.6. Management Implications

The field results have practical implications for plantation assessment and management. Competition indices such as Hegyi and BAL may help identify trees or microsites where radial growth is constrained. H/D and Δ *ln(H/D)* can serve as practical indicators of growth allocation and potential stem slenderness, especially when combined with repeated DBH and height measurements [13,14,15,16]. These indicators complement broader plantation assessment frameworks that consider productivity, resource use, and stand structure [5]. The center-edge comparison suggests that local competition management may be associated with radial growth and stand structure, although direct intervention experiments are needed before prescribing specific treatments.

Molecular markers and hub genes should be viewed as research tools and future screening candidates rather than immediate operational tools. Genomic and transcriptomic resources can support breeding-oriented research and biological interpretation, but their translation to operational forestry requires robust validation across genetic backgrounds and environments [6,42,44,52]. Transcriptomic data can help identify biological processes associated with growth allocation, but routine plantation management will likely require simpler, lower-cost indicators. Linking field competition metrics with robust molecular signatures may eventually improve genotype or site evaluation, but such applications require independent validation and clearer evidence of stability across environments.

## 4. Materials and Methods

### 4.1. Study Site and Plot Design

The study was conducted in Luoding City, Guangdong Province, China, in a managed *Eucalyptus urophylla* × *E. grandis* clone DH32-29 plantation area. The stands were planted in April 2022 at 2 m × 3 m spacing, corresponding to an initial density of approximately 1667 trees ha^−1^. The growth interval analyzed here used measurements from 2025 and 2026.

The study used 12 selected stand units within the plantation area with broadly comparable site and management conditions. Fixed plots were used to characterize interior trees and local neighborhood structure. Edge trees were sampled at the actual outer boundary of the plantation stands, rather than from fixed-plot boundaries. No fixed distance in meters from the boundary was used to define edge trees; their classification was based on their position at the true stand edge and the presence of open lateral growing space.

This study used an observational field design. The comparisons between interior and edge trees, and the analyses of neighborhood competition, were therefore interpreted as associations rather than direct evidence of causality. In particular, the neighborhood competition indices described below were used to characterize local stand structure and competitive heterogeneity; they were not direct measurements of light availability, soil moisture, nutrient supply, or wind exposure.

### 4.2. Center-Tree and Edge-Tree Definitions

Center trees were representative interior trees selected from fixed plots within the 12 selected stand units. Eligible center trees had complete records for DBH, total height, crown width, height to crown base, and spatial coordinates. Dead trees and trees with broken tops, visible disease, mechanical damage, wind throw, or other disturbance were excluded.

For neighborhood competition calculations, trees close to fixed-plot boundaries were not used as focal center trees to avoid incomplete neighbor information within the 3 m neighborhood radius. This technical exclusion is distinct from the ecological definition of edge trees, which were sampled from the true stand edge.

Edge trees were sampled at the actual outer boundary of the plantation stands, not from fixed-plot boundaries or from a meter-defined buffer. Eligible trees had at least one side without canopy obstruction by same-age *Eucalyptus* and sufficient lateral space for crown expansion. They were from the same clone and stand age and had broadly comparable management background as center trees. Trees adjacent to roads or canals, severe canopy gaps, or areas affected by disease, physical damage, or human disturbance were excluded.

### 4.3. Growth Indices and Allometric Analyses

Height-to-diameter ratio (H/D) was calculated as total height in meters divided by DBH in centimeters. Therefore, H/D was treated as a morphological index with mixed units, not as a dimensionless ratio.

To further evaluate how growth was allocated between tree height and stem diameter, the periodic height–diameter allometric growth coefficient *m_h,d_* was calculated from repeated measurements. This coefficient has been used to describe the proportional relationship between relative height growth and relative diameter growth, and can therefore serve as an indicator of growth allocation between vertical extension and radial thickening [36,53,54]. For consecutive measurement years, relative height growth and relative diameter growth were calculated as:

*RGR_H_* = *ln*(*H_t_*_2_) − *ln*(*H_t_*_1_)

*RGR_D_* = *ln*(*DBH_t_*_2_) − *ln*(*DBH_t_*_1_)

where *t1* and *t2* denote consecutive measurement years. The individual-level height–diameter allometric growth coefficient was calculated as:

$$m_{h,d}=\frac{{\mathrm{RGR}}_{H}}{{\mathrm{RGR}}_{D}}$$

When *m_h,d_* = 1, relative height growth and relative diameter growth are proportional, indicating isometric growth. When *m_h,d_* < 1, relative height growth is lower than relative diameter growth, indicating relatively stronger radial growth. When *m_h,d_* > 1, relative height growth exceeds relative diameter growth, indicating relatively stronger height-oriented growth. All 618 complete records in the analyzed 2025–2026 field dataset had positive *RGR_D_* values (minimum 0.0142); no zero denominators occurred, and no near-zero threshold was applied to exclude observations. The maximum observed *m_h,d_* was 13.56, so values from trees with small positive *RGR_D_* were retained and interpreted cautiously.

Population-level height–diameter allometric slopes were estimated separately from the individual-level growth coefficient using log–log allometric models, following common approaches for allometric line fitting and tree growth allocation analysis [13,55]. Specifically, mixed-effects models of log-transformed height and DBH were fitted as:

*log*(*H_ij_*) = *β*_0_ + *β*_1_*log*(*DBH_ij_*) + *b_j_* + *ε_ij_*

where *H_ij_* and *DBH_ij_* are the height and DBH of tree *i* in plot *j*, *β_1_* is the population-level allometric slope, *b_j_* is the random plot effect, and *ε_ij_* is the residual error. Models were fitted using the lme4 package in R [56].

### 4.4. Neighborhood Competition Indices

Neighborhood competition was quantified within a 3 m radius around each focal tree to include the immediate planted neighbors within approximately one inter-tree spacing along the 3 m planting axis (the stand spacing was 2 m × 3 m). This radius was used consistently for all focal trees; no radius-sensitivity analysis was conducted [14,19,20,21]. Four indices were calculated: the Hegyi competition index, basal area in larger trees (BAL), neighborhood tree number, and crown overlap ratio. These indices were used to characterize local competitive position and potential size-asymmetric competition among neighboring trees [17].

The Hegyi competition index was used to represent the combined effects of neighbor size and distance [19]:

$${\mathrm{Hegyi}}_{i}=\sum_{j=1}^{n_{i}}\frac{\frac{{\mathrm{DBH}}_{j}}{{\mathrm{DBH}}_{i}}}{d_{\mathrm{ij}}}$$

where *DBH_i_* is the DBH of focal tree *i*, *DBH_j_* is the DBH of neighbor tree *j*, *d_ij_* is the distance between the focal tree and neighbor tree, and *n_i_* is the number of neighbors within the 3 m radius.

BAL was calculated as the summed basal area of all neighboring trees within the 3 m radius that had larger DBH than the focal tree, following the concept of basal area in larger trees as an index of suppression by larger neighboring trees [20]. Neighborhood tree number was defined as the count of neighboring trees within the 3 m radius. Crown overlap ratio was calculated from the overlap between the focal tree crown projection and neighboring crown projections within the same neighborhood radius.

### 4.5. RNA Sequencing Sampling Design

RNA sequencing was conducted on 72 sampled trees (36 center and 36 edge trees), a subset of the full phenotypic dataset. In each of the 12 stand units, three trees per group were selected using a stratified growth-contrast approach rather than random sampling: center-tree candidates prioritized stronger neighborhood competition and higher H/D, whereas edge-tree candidates prioritized weaker lateral competition and lower H/D. For WGCNA, two sample-level outliers (C8_2 and R4_2) were excluded because they strongly affected the analysis results, leaving 70 samples in the expression matrix. The module–trait correlation output and this 70-sample matrix came from the same LC-Bio WGCNA workflow using the platform default settings.

Cambial tissue was collected from the sampled trees on 11 April 2025, between 09:00 and 11:00 for RNA sequencing. Total RNA was extracted using plant-specific protocols, and libraries were prepared and sequenced on the Illumina NovaSeq platform. Raw reads were quality-checked using FastQC and trimmed to remove adapters and low-quality bases. Clean reads were aligned to the *E. urophylla* × *E. grandis* reference genome [8] using HISAT2 [57], and transcript abundance was estimated with StringTie [58].

Differential expression was analyzed on the LC-Bio cloud platform using DESeq2 with the platform’s default analysis settings, separately for each of the 12 stand units by comparing the three edge RNA-seq samples with the three center samples from that unit [59]. Genes with |log2FC| > 1 and an adjusted q value < 0.05 were classified as differentially expressed.

### 4.6. Weighted Gene Co-Expression Network Analysis

Weighted gene co-expression network analysis (WGCNA), including the module–trait correlation output, was conducted as one workflow on the LC-Bio cloud platform using its default analysis settings. Two sample-level outliers (C8_2 and R4_2) were excluded because they strongly affected the results; the resulting input matrix contained 9778 genes and 70 RNA-seq samples. The exported report indicated a soft-thresholding power of 6, minModuleSize = 30, deepSplit = 2, and mergeCutHeight = 0.25. The report states that genes with expression below 10 in each of the 70 samples were filtered; it gives no separate variance cutoff. Module eigengenes were correlated with the seven traits present in the exported matrix (RATIO, DBH, HEIGHT, *m_h,d_*, *RGR_D_*, *RGR_H_*, and position) using Pearson correlation. Benjamini–Hochberg q values were recalculated across all 105 module–trait tests and are reported in Supplementary Table S2. Competition indices were not present in the exported module–trait matrix; no direct module–competition-index association is claimed.

A signed co-expression network was constructed. The soft-thresholding power was selected using chooseSoftThreshold to approximate scale-free topology [27]. Module detection used minModuleSize = 30, deepSplit = 2, and mergeCutHeight = 0.25.

Although neighborhood competition indices were measured in the field, they were not included in the available WGCNA module–trait export. Therefore, no module–competition-index correlations were estimated from this export or used for module selection.

### 4.7. Machine-Learning Feature Prioritization

XGBoost was used to prioritize predictors associated with individual-level allometric variation [29], with *m_h,d_* as the response variable. The preserved feature-importance output lists 175 predictors. The preserved evaluation output documents 49 training trees and 21 held-out test trees; test performance was summarized using *R*^2^, RMSE, and MAE. The available analysis records do not specify the feature-construction procedure, the rule or random seed used for the split, cross-validation, or hyperparameter tuning. We therefore report the saved test-set metrics without inferring undocumented validation or tuning procedures, and interpret the model as an associative feature-screening analysis rather than mechanistic evidence.

### 4.8. qRT-PCR Validation

qRT-PCR was performed for 12 candidate genes using three biological replicates per group and three technical replicates per sample. RNA quality and quantity were assessed by agarose gel electrophoresis, NanoDrop spectrophotometry, and Qubit fluorometry. Total RNA (1 μg) was reverse-transcribed using the Evo M-MLV RT Mix Kit with gDNA Clean for qPCR (Accurate Biology, AG11734). Amplification was performed in 20 μL reactions using ChamQ Universal SYBR qPCR Master Mix (Vazyme) on a LightCycler 480 II system (Roche). Cycling conditions were 95 °C for 30 s, followed by 40 cycles of 95 °C for 10 s and 60 °C for 30 s. Melting-curve analysis and no-template controls were included. Primer sequences are provided in Supplementary Table S4. Relative expression was calculated using the 2^−ΔΔCt^ method, and center-versus-edge log_2_ fold changes were compared with RNA-seq estimates using Pearson and Spearman correlations. These assays assessed expression trends, not gene function, with reference to qRT-PCR reporting considerations [30].

### 4.9. Statistical Analysis

Spearman rank correlations were used to assess monotonic associations between the four neighborhood competition indices and diameter and height increments; a normality test was not used to select the correlation method. Benjamini–Hochberg adjustment was applied across these eight competition–growth correlations. Center–edge differences were first tested using two-sided Mann–Whitney U tests. To assess sensitivity to clustering within the 12 stand units, we also fitted linear models with group and stand unit as fixed effects and calculated standard errors clustered by stand unit; cluster-adjusted 95% confidence intervals and *p* values used 11 degrees of freedom. Benjamini–Hochberg adjustment was applied across the six field traits in this sensitivity analysis, which is reported in Supplementary Table S5 alongside group medians, interquartile ranges, and sample sizes. Pearson correlations were used for WGCNA module–trait associations, with Benjamini–Hochberg adjustment across the 105 tests in the exported matrix. Pearson and Spearman correlations were used to assess concordance between RNA-seq and qRT-PCR log_2_ fold changes across the 12 candidate genes. Spatial autocorrelation was not assessed.

## 5. Conclusions

Competition indices were negatively associated with diameter and height increments in the field dataset. Unadjusted center–edge comparisons suggested broader differences in growth traits, but sensitivity analyses accounting for stand unit supported only the differences in diameter increment and H/D after correction across six traits. Because the study is observational and edge positions may also differ in light, wind exposure, microclimate, and soil conditions, these results describe associations and do not isolate neighborhood competition as a cause.

WGCNA identified co-expression modules with associations to individual allometric growth traits. After correction for multiple testing, MEmagenta was associated with *m_h,d_* and *RGR_H_*; its association with *RGR_D_* was nominal only. XGBoost provided moderate test-set performance and helped prioritize candidate expression features, while qRT-PCR supported expression trends for selected genes but did not validate gene function. These findings indicate associations among neighborhood structure, growth patterns, and gene-expression variation, without establishing causal links.

Further work should incorporate direct environmental measurements, repeated temporal sampling, independent-site validation, and functional experiments. Such studies are needed before making causal claims about candidate genes or applying gene-informed growth models in operational plantation management.

## Data Availability

The data presented in this study are available in the article and its Supplementary Materials. Raw RNA-seq data are available from the corresponding author upon reasonable request.

## Supplementary Materials

The following supporting information can be downloaded at: https://www.mdpi.com/article/10.3390/plants15213341/s1, Table S1: Unit-specific differential expression results; Table S2: WGCNA module–trait correlations and paired RNA-seq/qRT-PCR expression estimates; Table S3: Competition–growth correlations; Table S4: qRT-PCR primer sequences; Table S5: Plot-adjusted center–edge sensitivity analyses.
